# Every Word and Action: Using Cinemeducation to Enhance Medical Students’ Awareness of and Upstander Responses to Disability Microaggressions

**DOI:** 10.15766/mep_2374-8265.11626

**Published:** 2026-08-14

**Authors:** Krishna Mohan Surapaneni

**Affiliations:** 1 Professor, Department of Biochemistry, Panimalar Medical College Hospital and Research Institute; Head and Professor, Department of Medical Education, Panimalar Medical College Hospital and Research Institute

**Keywords:** Microaggressions, Disability Inclusion, Visual Storytelling, Upstander Behavior, Implicit Bias, Advocacy, Reflective Practice, Empathy, Communication Skills, Competency-Based Medical Education, Ethics/Bioethics

## Abstract

**Introduction:**

Medical students are rarely given structured opportunities to explore how persons with disabilities (PwD) experience subtle discrimination, especially in clinical or academic settings. Despite the inclusion of disability-related competencies in medical school curriculum, microaggressions remain insufficiently addressed. This activity sought to create a reflective, immersive learning space to sensitize first-year medical students to microaggressions against PwD through a 2-day cinemeducation-based intervention.

**Methods:**

One hundred fifty first-year medical students participated in two 3-hour faculty-facilitated small-group activities using curated film clips aligned with 12 identified themes of microaggressions against PwD. Each session combined film viewing, self-reflection, and structured group dialogues. Students maintained personal diaries for 6 months, which are long-term reflective documentation tools, to document their awareness, responses, and behavioral changes. Mixed-methods evaluation included pre-/posttests, confidence rating scales, session feedback, and semistructured interviews.

**Results:**

Learners demonstrated improvement in recognizing microaggressions, articulating inclusive responses, and confidence as an upstander (*P* < .0001), suggesting early gains in awareness and response. Thematic analysis revealed learners’ increased self-awareness, empathy, and willingness to act as upstanders. Students reported shifts in language use, peer education efforts, and personal growth. The diaries served as a sustained reflective tool that reinforced students’ commitments to inclusive practice.

**Discussion:**

This activity effectively bridged affective and cognitive learning. Moving beyond lecture-based instruction invited students to engage with the lived experiences of disability in a humanized and emotionally resonant manner. Cinemeducation, when embedded within a longitudinal reflective framework, holds transformative potential to promote disability inclusion and ethical awareness in medical education.

## Educational Objectives

By the end of this session, students will be able to:

1. Describe the term “microaggressions” and the various forms of microaggressions against persons with disabilities.
2. Discuss critically on curated movie clips that depict subtle discriminatory behaviors and their emotional and social impact on individuals with disabilities.
3. Reflect on everyday microaggressions happening in academic and clinical learning environments.
4. Journal the actions taken to stop or prevent microaggressions during the 6-month “Change” period.
5. Improve their confidence in standing up against microaggression and demonstrate commitment to disability inclusion.

## Introduction

Microaggressions are everyday interactions or actions that are subtle yet discriminatory or demeaning toward marginalized groups of individuals.^1^ Regrettably, such microaggressions are common against persons with disabilities (PwD). Although these derogatory actions may be unintended at times, they convey more than just disrespect; they can potentially perpetuate stigma, promote ableism, reinforce harmful stereotypes, and foster an environment that feels noninclusive and alienating.^2^ Unlike other forms of discrimination that are often anchored in identity, disability-related microaggressions frequently intersect with assumptions about dependence, competence, and functional ability, making them uniquely embedded within clinical interactions.^3^ Over time, the cumulative effect of these microaggressions starts significantly impacting the sense of belonging, psychological safety, and overall well-being of individuals with disabilities. Further, vulnerability in health care is not limited to disability alone; individuals who are acutely unwell may temporarily occupy a devalued “sick role,” which can similarly expose them to subtle forms of disregard or marginalization within clinical settings.^4^ It is critical to start incorporating the teachings on microaggressions early in medical education, as these formative years of training lay the foundations of clinical practice and foster medical students to be more empathetic, equitable, and respectful of human dignity.^5,6^

Recently, greater attention has been given to addressing microaggressions in academic and clinical settings, particularly against PwD, lesbian, gay, bisexual, transgender, queer, intersex, asexual, and all inclusive (LGBTQIA+), and/or racial discriminations.^7–9^ These interventions have been particularly helpful in raising awareness and encouraging upstander behaviors among medical students and create a safer inclusive learning environment.^10^ Additionally, *MedEdPORTAL* has contributed significantly to the growing body of literature on training residents and faculty to address microaggressions using varied approaches, including interactive workshops, standardized patients, simulations, and practical toolkits.^11–13^ While these trainings were found to be efficacious, the authors did not analyze the long-term impact or students’ self-reflection of their changed behaviors and upstander actions. An additional common approach is the use of paper- and simulation-based training, with cases and standardized interactions, to raise awareness of microaggressions. While these methods offer structured and controlled learning environments, they might fall short of capturing the emotional depth that microaggressions carry. It must be emphasized that the impact of microaggressions are not just cognitive learning points but deeply felt experiences, often leaving lasting impressions on those on the receiving end. Given the affective dimension of such encounters, the use of cinema, known for its profound capacity to evoke empathy and emotional engagement, presents a potentially powerful pedagogical strategy.^14,15^

Evidence for the efficacy of cinemeducation in undergraduate medical education is limited. However, literature on exploring student preferences shows media as consistently rated highly given its instant ability to capture attention and leave a strong impact.^16^ This educational activity demonstrates how cinemeducation can be used as an effective pedagogical approach to enhance medical students’ awareness, confidence, and willingness to engage in upstander behavior in response to microaggressions, particularly toward PwD. To do so, this educational experience is not delivered in a single session but is longitudinal and draws on principles of experiential learning and reflective practice,^17,18^ including the MAP (Monitor–Act–Progress) diary to support students’ ongoing reflection on what they observed, how they responded, and how their thinking and actions evolved over time.

## Methods

### Educational Settings and Learners

This curriculum was implemented at Panimalar Medical College Hospital and Research Institute, a private medical college in South India. At the time of implementation, the undergraduate curriculum did not include formal instruction on microaggressions or their impact. Although disability inclusion was addressed through a broader competency, students had limited exposure to the concept of subtle discrimination and its effects on individuals with disabilities.

The curriculum was delivered during the 1-month foundation course that occurs at the start of the first professional year and aims to orient students to the values and expectations of the medical profession. Thus, no formal prerequisite knowledge or training was expected from learners.

A total of 150 first-year medical students participated. Students provided written informed consent and were allowed to opt out at any time they felt uncomfortable continuing. The study received ethical clearance from the Institutional Human Ethics Committee of Panimalar Medical College Hospital and Research Institute (Approval No. PMCHRI-IHEC-203, August 2024).

### Faculty Preparation

The activity was conducted in small groups facilitated by faculty volunteers. Facilitators were faculty members involved in AETCOM (Attitudes, Ethics, and Communication) sessions at our institution, and were comfortable facilitating these discussions on professional values and behaviors.

Prior to implementation, facilitators were oriented to the objectives of the session and guided to promote meaningful, respectful, inclusive, and participatory group discussions (Appendix A). The lead investigator led the facilitators through a 60- to 90-minute interactive session that included clip-watching and a briefing on discussion points prior to implementation.

### Curriculum Development

A literature search was conducted to identify common forms of microaggressions experienced by PwD. Twelve themes were identified. The themes included (1) Assuming Incompetence, (2) Patronizing Language, (3) Exclusion, (4) Offering Rights, (5) Overt Inspiration, (6) Accessibility as an Afterthought, (7) Assuming Disability is Always Visible, (8) Dismissive Comments, (9) Overgeneralization, (10) Excessive Sympathy, (11) Intrusive Questions, and (12) Speaking Over. Descriptions for each theme are provided in Appendix B. All clips were selected from publicly accessible YouTube videos. Additionally, all clips were in English except for 2, which had English subtitles (Appendix C). The clips were chosen to stimulate discussion regarding the causes, emotional impact, consequences, and potential responses to disability-related microaggressions.

### Curriculum Implementation

This curriculum was delivered over 2 consecutive days (October 27–28, 2024) with two 3-hours sessions on each day. Students were randomly assigned to 30 small groups of 5 learners with a facilitator assigned to each group.

To begin the first session, students completed a pretest with 15 scenario-based, open-ended questions aligned with the microaggression themes (Appendix D). Responses were scored independently by 2 faculty members using a 3-point rubric, and mean scores were used for analysis.

All facilitators used a common PowerPoint presentation. The presentation included session learning objectives, types of disabilities, an overview of microaggressions and their impact, an introduction to cinemeducation, the curated film clips, reflection questions, and explanations of the 12 themes (Appendix E). Facilitators also were given a guide that described each film clip, its associated themes, and discussion points prior to the session (Appendix F).

Six themes were addressed each day. Approximately 30 minutes were allocated to each theme, including for film-watching, self-analysis, and group discussion. Each clip time ranged between 4 and 6 minutes. Following each clip, learners completed a structured reflection worksheet (Appendix G) to support their personal analysis. The worksheets were used to facilitate immediate personal reflection and to initiate group discussions during the session; they were not collected for analysis.

After viewing each clip, learners engaged in facilitated small-group discussions focused on identifying the microaggression, exploring its underlying assumptions, considering its emotional and practical impact on people with disabilities, and discussing potential responses and strategies for promoting inclusion.

After the cinemeducation activity, the evaluation approach followed the Kirkpatrick model.^19^ At the conclusion of day 2, students completed the posttest, which used the same scenario-based questions as the pretest (see Appendix D; level 2) to assess changes in their ability to recognize and interpret microaggressions. Students also completed a 12-item validated questionnaire on a 5-point Likert scale to obtain learner reaction and feedback on the session (level 1) (Appendix H). Content validity for the questionnaire was established through review by 20 medical education faculty, face validity was assessed through pilot testing with 24 medical students from a different cohort, and internal consistency was confirmed using Cronbach's alpha (0.86).

Following completion of the curriculum, students received a MAP diary (Appendix I). Over the subsequent 6 months, students were encouraged to observe their own and others’ behavior in academic and clinical settings. Behavior tracking began immediately after the session and continued for 6 months (level 3). Students were encouraged to voice out against microaggressions, teach a peer when they commit one, and commit themselves to inclusive language and behavior. This was called the “Change” period. Students finally reflected on these and journaled the actions they took to stop or prevent microaggressions. During this 6-month “Change” period, students were encouraged to engage in continuous reflection and to make regular entries in their MAP diaries, ideally weekly or whenever they encountered or reflected on a relevant experience. While a fixed number of entries was not mandated, students were expected to document meaningful observations, actions, and reflections consistently over time.

**Figure 1.**
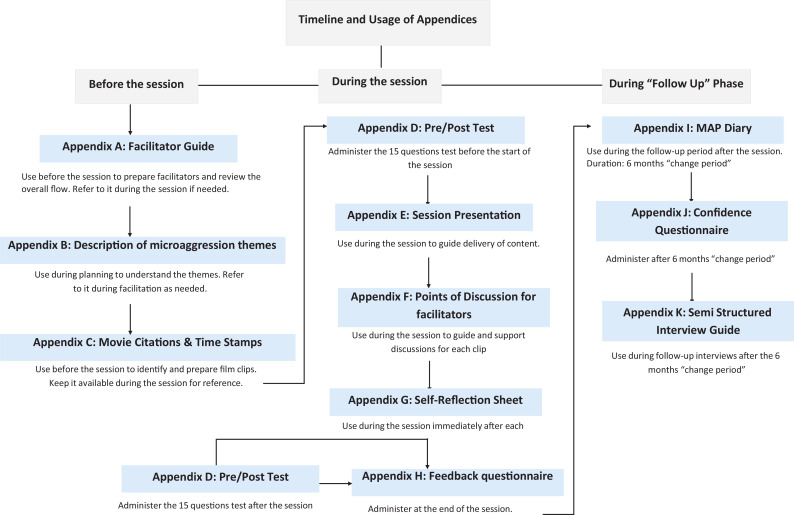
A schematic overview of the timeline and usage of appendices.

Six months after the activity, students submitted their MAP diaries and completed a 12-item online questionnaire (Appendix J) assessing confidence as upstanders before and after participation. Additionally, semistructured one-on-one or small group interviews were conducted, based on students’ preferences, to explore changes in learners’ experiences and perceived behavioral changes related to recognizing and addressing disability-related microaggressions (Appendix K). A schematic overview of the timeline and usage of appendices is shown in Figure 1.

### Analysis

Quantitative data were analyzed using Stata/BE version 18.5 with statistical significance set at *P* < .05.

Continuous variables were summarized using univariate statistics to report means and standard deviations. Group differences were assessed using *t* tests and ANOVA for normally distributed data. For nonnormally distributed data, Mann-Whitney U and Kruskal-Wallis tests were used.

Qualitative data were collected from semistructured interviews conducted 6 months after the intervention with a purposive sample of students (*N* = 109; 62.3% female, 36.6% male). Out of 109 students, 20 participated in one-on-one interviews lasting for 20 minutes each and 89 students participated in 17 small-group interviews (5 students per group; 30 minutes each). To further examine learners’ experiences over time, the MAP diary submissions were analyzed as longitudinal reflective data and considered alongside interview data. Interviews were conducted by trained faculty members with experience in qualitative methods who were not involved in delivering the curriculum. Given the sensitive nature of the topic, this minimized social desirability bias and encouraged open, honest responses.

Interview recordings were audio-recorded, transcribed verbatim and analyzed using Braun and Clarke's 6-phase thematic analysis approach.^20^ Both formats used the same interview guide. One-on-one interviews were conducted to provide more space for students willing to share additional information. Transcripts from both individual and small-group interviews were analyzed together to identify common patterns and shared experiences. Three data analyzers experienced in qualitative research and not involved in the project familiarized themselves with the data through repeated reading, generated initial codes inductively, and organized them into themes, which were reviewed, refined, and defined to reflect patterns across participants. Discrepancies in coding were resolved through consensus, with additional reviewers consulted when needed. Member checking was performed by clarifying interpretations with participants where necessary.

## Results

A total of 150 students participated in this activity, of which 64.4% were female. The mean (standard deviation) age of the students was 19.2 years (1.8). The response rate for the pre/posttest was 96.6% (*n* = 145). The mean pretest score was 10.1 (2.4) out of 30, and the posttest score was 22.3 (2.8), indicating positive progression in learners’ understanding and response to microaggressions (*P* < .0001).

**Figure 2.**
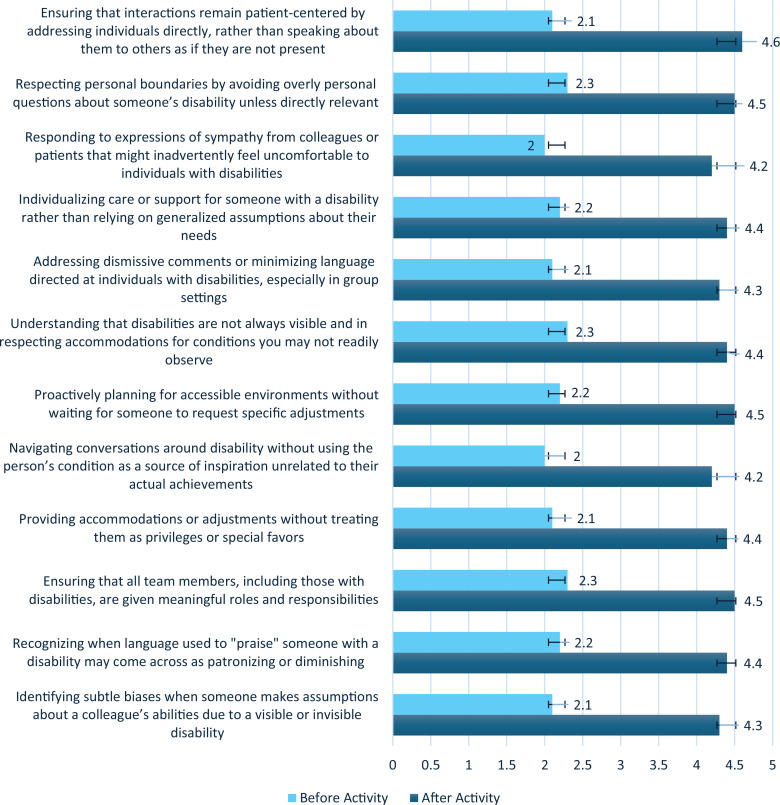
Comparison of student confidence level (*n* = 142).

Postactivity session evaluations were returned by 97.8% of participants and revealed consistently high satisfaction. Students reported the session as clear, well structured, and personally meaningful. Students’ responses for the survey items with mean score are given in Table 1. Students reported a statistically significant increase in confidence across all 12 assessed domains following the cinemeducation intervention (*P* < .0001) with 94.6% (*n* = 142) students returning the survey. Detailed pre- and postactivity confidence scores for each domain are presented in Figure 2.

**Table 1.**
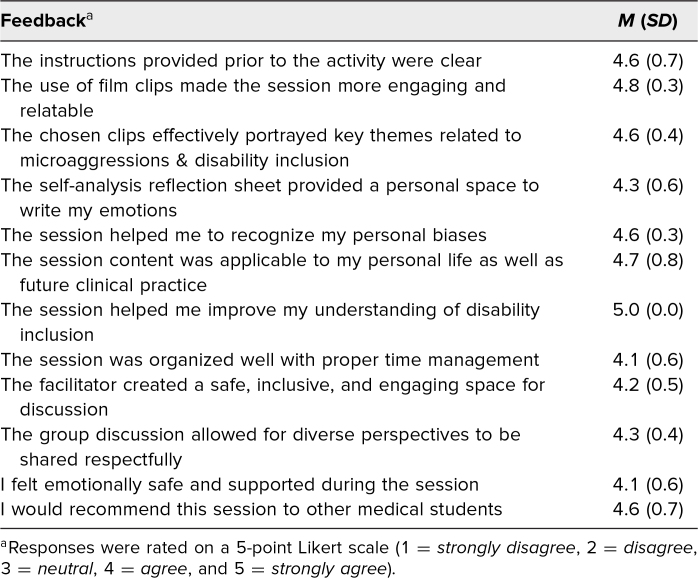
Student Feedback (*n* = 147)

After the 6-month “Change” period, all students submitted their MAP diaries. The reflections revealed meaningful engagement with the session's core objectives, indicating that the activity extended beyond passive learning to facilitate active internalization of concepts. In the “Monitor” section, students demonstrated heightened awareness of microaggressions, often reflecting on how their perception of everyday interactions had evolved over the 6-month period. Many reported increased sensitivity to subtle forms of bias in academic and clinical environments and acknowledged their own areas for growth. The “Act” segment highlighted the adoption of personal strategies such as using inclusive language, addressing inappropriate remarks, and mentoring peers through informal conversations. Notably, several learners articulated concrete short-term goals to foster more equitable and respectful environments. In the “Progress” section, students described moments where they had intervened or modeled inclusive behavior, as well as challenges encountered in confronting microaggressions, particularly in hierarchical settings. Despite such barriers, many noted positive shifts in peer and faculty receptivity.

Interviews revealed 7 key areas of transformation in students’ perspectives and behavior following the cinemeducation sessions. First, students spoke about the emotional impact of the films, with many describing how certain scenes stayed with them long after the session, making the issues feel “real” and deeply personal. Second, they reported a growing awareness of microaggressions, often sharing that they now recognized subtle biases in tone, language, and even silence that they had previously overlooked. A third theme emerged around the recognition of personal bias, in which learners reflected candidly on moments of discomfort, acknowledging that they had unknowingly contributed to microaggressions in the past and expressing a commitment to change. The value of peer discussions was another prominent theme, as students found the small-group setting a safe space to listen, share, connect, and learn from diverse perspectives. Many students described moments when a classmate's story shifted their thinking entirely and also having taken action since the session, marking the emergence of upstander identity. Whether it was correcting a peer, advocating for inclusive language, or pushing for accessibility, these experiences reflected a growing sense of accountability. The MAP diary played a key role in reinforcing this journey; it helped students remain reflective, track their growth, and stay mindful in day-to-day interactions. Lastly, there was a strong and consistent support for curricular integration, with students voicing that such emotionally engaging and reflective learning must not remain optional or peripheral but should be embedded into the heart of medical education. Representative comments for each theme are presented in Table 2.

**Table 2.**
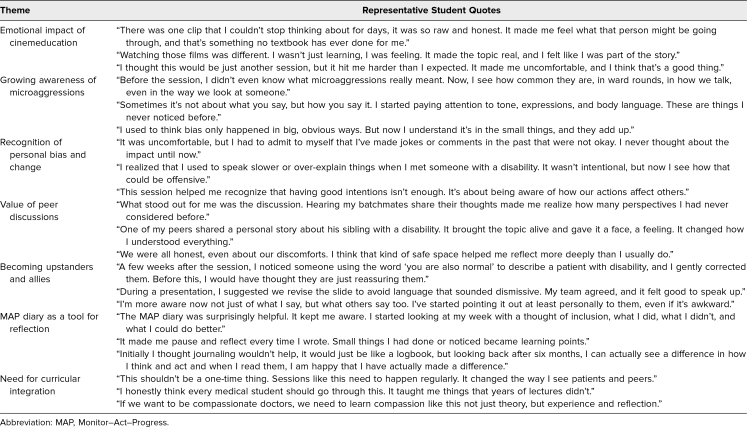
Thematic Analysis

## Discussion

This educational innovation marked a deliberate and impactful shift in how undergraduate medical students are introduced to microaggressions against PwD. Through a multimodel staged pedagogical design integrating curated film clips, guided reflections, small-group discussions, and longitudinal self-monitoring, the program offered learners not just knowledge but a sustained opportunity for transformation in attitudes and behaviors.

The innovation accomplished 3 major educational goals. First, it sensitized learners to forms of discrimination that often operate below the threshold of conscious recognition, which are subtle, everyday instances that PwD routinely experience. These are not traditionally foregrounded in medical training, yet they profoundly shape health care interactions. Second, the intervention facilitated learners’ capacity to internalize these understandings and translate them into reflective, empathetic responses. The deliberate use of visual storytelling, anchored in practical themes, catalyzed emotional engagement that passive instruction rarely achieves. Third, the activity fostered behavioral intent and accountability through the MAP diary, which extended learning into lived experiences over 6 months. This sustained dimension offered a unique longitudinal scaffold for ethical growth.

The development and implementation of this activity were grounded in intentional instructional design. Unlike conventional teaching strategies that center on lectures or theoretical frameworks, film, as a medium, offered a visceral immediacy that helped surface implicit assumptions and affective responses. Evaluation of the intervention indicated conceptual maturation, as learners moved from vague, generalized views of disability inclusion to more nuanced, context-aware understandings. They began identifying tone, language, and implicit bias with increasing sophistication. In interviews and diary entries, students described situations in which they recognized ableist language in academic discourse, respectfully corrected peers, or advocated for accessibility in daily routines. These narratives provide compelling qualitative evidence of learning transfer that is a key marker of impactful education.

The project yielded significant educational value; however, several limitations must be acknowledged. This intervention was conducted at a single institution involving medical students in the early stages of training. While this allowed engagement at a formative point, it may also limit how far these findings can be applied to more clinically experienced learners or to different institutional settings. The use of terms such as “microaggressions” and “upstander behavior” helped structure the sessions, but these may not resonate equally with all learners. In practice, anchoring these ideas in lived examples and discussion appeared to make them more accessible. The effectiveness of the sessions also depended on facilitation and on students’ willingness to engage with the material. As the program is scaled, differences in facilitation may influence how consistently it is delivered.

An additional consideration relates to the assessment approach used. The pre- and posttest scenarios were intended to assess learners’ understanding of disability-related microaggressions and their ability to respond appropriately to situations encountered in academic and clinical environments. Future iterations could further strengthen assessment validity by incorporating less explicitly signposted scenarios that require learners to independently identify the underlying microaggression theme and propose an appropriate response.

Another limitation pertains to the longitudinal follow-up mechanism. While the MAP diary encouraged sustained engagement, it was still a self-report tool and potentially subject to social desirability bias. Although learner feedback was largely positive, the possibility of discomfort, resistance, or socially desirable responding cannot be excluded, particularly given the reflective and affective nature of the sessions; such responses may represent an integral part of the learning process and warrant more explicit exploration in future iteration. Nonetheless, the depth of entries and triangulation with interview data could suggest sincerity and reflective maturity among respondents. Future iterations may benefit from integrating observation-based assessments or multisource feedback to strengthen the evidence base.

Despite these challenges, the implications of this work are broad. First, it establishes a replicable model for introducing complex sociocultural constructs, such as microaggressions, through an emotionally resonant and cognitively structured format. Second, it positions disability inclusion not as an optional or peripheral conversation but as a core element of professional identity formation. This compels educators to recognize that microaggressions can erode trust and equity in clinical spaces and that addressing them requires more than awareness; it requires capacity-building in real contexts. Some of the clips clearly stayed with students, which was noticeable in how they spoke about them later. That said, it is hard to separate whether this was because of the medium itself or the way those particular scenes were put together. It probably points more to how these situations were made visible and discussed rather than the clips alone. In that sense, similar responses might still be possible with other clips that bring out the same issues.

Such flexibility empowers educators and learners to incorporate culturally relevant narratives and personal resonance, fostering choice and richer engagement. This activity could be adapted and embedded within longitudinal professionalism tracks, early clinical exposure modules, and interprofessional education initiatives. The MAP diary, in particular, could serve as a versatile reflection tool across various domains of hidden curriculum, from gender equity to caste and cultural sensitivity.

Future work should explore adapting this activity across languages and educational settings through multi-institutional implementation. There is also a need for validated tools to assess the clinical application of inclusive practices and to evaluate outcomes beyond Kirkpatrick level 3. This may include patient-reported experiences, multisource feedback, and structured observation of clinical interactions. Additionally, faculty development initiatives should focus on sustaining inclusive practices through mentorship and role modeling.

## Appendices


Facilitator Guide.pptxMicroaggression Themes.docxMovie Citations and Time Stamps.docxPre- and Posttest.docxSession Presentation.pptxPoints for Discussion.docxSelf-Reflection Sheet.docxFeedback Questionnaire.docxMAP Diary.docxConfidence Questionnaire.docxSemi-Structured Interview Prompts.docx
*All appendices are peer reviewed as integral parts of the Original Publication.*
