# Supplementary material for: Every Word and Action: Using Cinemeducation to Enhance Medical Students’ Awareness of and Upstander Responses to Disability Microaggressions
Source: MedEdPORTAL. 2026 Aug 14;22:11626. doi: 10.15766/mep_2374-8265.11626 (PMC13473049; doi:10.15766/mep_2374-8265.11626)
Supplement: Supplementary file 1 [file mep_2374-8265.11626-s001.zip › I. MAP Diary.docx]

**MAP (Monitor, Act & Progress) Diaries:**

**A Reflective Journal on**

**Microaggression**

**Awareness and Action**

**In Academic & Clinical Settings**

**Name:**

**Date:**

**Instructions**

As part of your commitment to fostering an inclusive and respectful medical environment, this MAP (Monitor, Act, Progress) Diary serves as a tool for you to reflect on your journey in recognizing and addressing microaggressions.

In the past six months, you have engaged with cinemeducation, observed real-world scenarios, and taken steps toward creating a more inclusive medical space. Now, this diary is an opportunity to pause and reflect on your experiences, the challenges you faced, and the progress you have made.

**You will be asked to document your reflections on:**

**Monitor: How your awareness of microaggressions has evolved and any incidents you have observed or addressed.**

**Act: The strategies you implemented, moments where you took action, and any personal goals you set or want to set.**

**Progress: The impact of your efforts, the responses from peers and faculty, and how these experiences have shaped your clinical practice.**

There are no right or wrong answers, this is your personal space to honestly assess your growth, recognize your efforts, and identify areas for continued improvement.

Your reflections will not only help solidify your learning but also contribute to shaping more effective interventions for future medical training.

**Take this moment to look back and write openly about your journey.**

Date:

**MONITOR (Awareness & Observations)**

1. **How has your awareness of microaggressions evolved over the past months? (Reflect on any new insights, realizations, or knowledge gained.)**
2. **Have you encountered microaggressions in clinical settings or academic discussions? (Describe specific incidents—what happened, and what impact it had.)**
3. **Have you recognized any microaggressive behaviors in yourself that you have worked to change? (Yes/No, If yes, describe the change.)**

**ACT (Personal Strategies & Goals)**

1. **What strategies have you used in the past months to address or mitigate microaggressions?**
2. **Have you set any personal goals if not set 2-3 new personal goals for the next three months on how to aim to eliminate microaggressions.**
3. **Have you started mentoring or guiding others in recognizing and addressing microaggressions? (Yes/No, If yes, describe how.)**

**PROGRESS (Reflection & Future Growth)**

1. **What has been the most effective change or action you have taken in the past months? (Describe a success story or moment of impact.)**
2. **What challenges or barriers have you faced in addressing microaggressions?**
3. **How have faculty or peers responded to your efforts? Have you noticed any shifts in their attitudes or behaviors?**

Date:

**MONITOR (Awareness & Observations)**

4. **How has your awareness of microaggressions evolved over the past months? (Reflect on any new insights, realizations, or knowledge gained.)**
5. **Have you encountered microaggressions in clinical settings or academic discussions? (Describe specific incidents—what happened, and what impact it had.)**
6. **Have you recognized any microaggressive behaviors in yourself that you have worked to change? (Yes/No, If yes, describe the change.)**

**ACT (Personal Strategies & Goals)**

4. **What strategies have you used in the past months to address or mitigate microaggressions?**
5. **Have you set any personal goals if not set 2-3 new personal goals for the next three months on how to aim to eliminate microaggressions.**
6. **Have you started mentoring or guiding others in recognizing and addressing microaggressions? (Yes/No, If yes, describe how.)**

**PROGRESS (Reflection & Future Growth)**

4. **What has been the most effective change or action you have taken in the past months? (Describe a success story or moment of impact.)**
5. **What challenges or barriers have you faced in addressing microaggressions?**
6. **How have faculty or peers responded to your efforts? Have you noticed any shifts in their attitudes or behaviors?**

Date:

**MONITOR (Awareness & Observations)**

7. **How has your awareness of microaggressions evolved over the past months? (Reflect on any new insights, realizations, or knowledge gained.)**
8. **Have you encountered microaggressions in clinical settings or academic discussions? (Describe specific incidents—what happened, and what impact it had.)**
9. **Have you recognized any microaggressive behaviors in yourself that you have worked to change? (Yes/No, If yes, describe the change.)**

**ACT (Personal Strategies & Goals)**

7. **What strategies have you used in the past months to address or mitigate microaggressions?**
8. **Have you set any personal goals if not set 2-3 new personal goals for the next three months on how to aim to eliminate microaggressions.**
9. **Have you started mentoring or guiding others in recognizing and addressing microaggressions? (Yes/No, If yes, describe how.)**

**PROGRESS (Reflection & Future Growth)**

7. **What has been the most effective change or action you have taken in the past months? (Describe a success story or moment of impact.)**
8. **What challenges or barriers have you faced in addressing microaggressions?**
9. **How have faculty or peers responded to your efforts? Have you noticed any shifts in their attitudes or behaviors?**

Date:

**MONITOR (Awareness & Observations)**

10. **How has your awareness of microaggressions evolved over the past months? (Reflect on any new insights, realizations, or knowledge gained.)**
11. **Have you encountered microaggressions in clinical settings or academic discussions? (Describe specific incidents—what happened, and what impact it had.)**
12. **Have you recognized any microaggressive behaviors in yourself that you have worked to change? (Yes/No, If yes, describe the change.)**

**ACT (Personal Strategies & Goals)**

10. **What strategies have you used in the past months to address or mitigate microaggressions?**
11. **Have you set any personal goals if not set 2-3 new personal goals for the next three months on how to aim to eliminate microaggressions.**
12. **Have you started mentoring or guiding others in recognizing and addressing microaggressions? (Yes/No, If yes, describe how.)**

**PROGRESS (Reflection & Future Growth)**

10. **What has been the most effective change or action you have taken in the past months? (Describe a success story or moment of impact.)**
11. **What challenges or barriers have you faced in addressing microaggressions?**
12. **How have faculty or peers responded to your efforts? Have you noticed any shifts in their attitudes or behaviors?**

Date:

**MONITOR (Awareness & Observations)**

13. **How has your awareness of microaggressions evolved over the past months? (Reflect on any new insights, realizations, or knowledge gained.)**
14. **Have you encountered microaggressions in clinical settings or academic discussions? (Describe specific incidents—what happened, and what impact it had.)**
15. **Have you recognized any microaggressive behaviors in yourself that you have worked to change? (Yes/No, If yes, describe the change.)**

**ACT (Personal Strategies & Goals)**

13. **What strategies have you used in the past months to address or mitigate microaggressions?**
14. **Have you set any personal goals if not set 2-3 new personal goals for the next three months on how to aim to eliminate microaggressions.**
15. **Have you started mentoring or guiding others in recognizing and addressing microaggressions? (Yes/No, If yes, describe how.)**

**PROGRESS (Reflection & Future Growth)**

13. **What has been the most effective change or action you have taken in the past months? (Describe a success story or moment of impact.)**
14. **What challenges or barriers have you faced in addressing microaggressions?**
15. **How have faculty or peers responded to your efforts? Have you noticed any shifts in their attitudes or behaviors?**

Date:

**MONITOR (Awareness & Observations)**

16. **How has your awareness of microaggressions evolved over the past months? (Reflect on any new insights, realizations, or knowledge gained.)**
17. **Have you encountered microaggressions in clinical settings or academic discussions? (Describe specific incidents—what happened, and what impact it had.)**
18. **Have you recognized any microaggressive behaviors in yourself that you have worked to change? (Yes/No, If yes, describe the change.)**

**ACT (Personal Strategies & Goals)**

16. **What strategies have you used in the past months to address or mitigate microaggressions?**
17. **Have you set any personal goals if not set 2-3 new personal goals for the next three months on how to aim to eliminate microaggressions.**
18. **Have you started mentoring or guiding others in recognizing and addressing microaggressions? (Yes/No, If yes, describe how.)**

**PROGRESS (Reflection & Future Growth)**

16. **What has been the most effective change or action you have taken in the past months? (Describe a success story or moment of impact.)**
17. **What challenges or barriers have you faced in addressing microaggressions?**
18. **How have faculty or peers responded to your efforts? Have you noticed any shifts in their attitudes or behaviors?**

Date:

**MONITOR (Awareness & Observations)**

19. **How has your awareness of microaggressions evolved over the past months? (Reflect on any new insights, realizations, or knowledge gained.)**
20. **Have you encountered microaggressions in clinical settings or academic discussions? (Describe specific incidents—what happened, and what impact it had.)**
21. **Have you recognized any microaggressive behaviors in yourself that you have worked to change? (Yes/No, If yes, describe the change.)**

**ACT (Personal Strategies & Goals)**

19. **What strategies have you used in the past months to address or mitigate microaggressions?**
20. **Have you set any personal goals if not set 2-3 new personal goals for the next three months on how to aim to eliminate microaggressions.**
21. **Have you started mentoring or guiding others in recognizing and addressing microaggressions? (Yes/No, If yes, describe how.)**

**PROGRESS (Reflection & Future Growth)**

19. **What has been the most effective change or action you have taken in the past months? (Describe a success story or moment of impact.)**
20. **What challenges or barriers have you faced in addressing microaggressions?**
21. **How have faculty or peers responded to your efforts? Have you noticed any shifts in their attitudes or behaviors?**

Date:

**MONITOR (Awareness & Observations)**

22. **How has your awareness of microaggressions evolved over the past months? (Reflect on any new insights, realizations, or knowledge gained.)**
23. **Have you encountered microaggressions in clinical settings or academic discussions? (Describe specific incidents—what happened, and what impact it had.)**
24. **Have you recognized any microaggressive behaviors in yourself that you have worked to change? (Yes/No, If yes, describe the change.)**

**ACT (Personal Strategies & Goals)**

22. **What strategies have you used in the past months to address or mitigate microaggressions?**
23. **Have you set any personal goals if not set 2-3 new personal goals for the next three months on how to aim to eliminate microaggressions.**
24. **Have you started mentoring or guiding others in recognizing and addressing microaggressions? (Yes/No, If yes, describe how.)**

**PROGRESS (Reflection & Future Growth)**

22. **What has been the most effective change or action you have taken in the past months? (Describe a success story or moment of impact.)**
23. **What challenges or barriers have you faced in addressing microaggressions?**
24. **How have faculty or peers responded to your efforts? Have you noticed any shifts in their attitudes or behaviors?**

Date:

**MONITOR (Awareness & Observations)**

25. **How has your awareness of microaggressions evolved over the past months? (Reflect on any new insights, realizations, or knowledge gained.)**
26. **Have you encountered microaggressions in clinical settings or academic discussions? (Describe specific incidents—what happened, and what impact it had.)**
27. **Have you recognized any microaggressive behaviors in yourself that you have worked to change? (Yes/No, If yes, describe the change.)**

**ACT (Personal Strategies & Goals)**

25. **What strategies have you used in the past months to address or mitigate microaggressions?**
26. **Have you set any personal goals if not set 2-3 new personal goals for the next three months on how to aim to eliminate microaggressions.**
27. **Have you started mentoring or guiding others in recognizing and addressing microaggressions? (Yes/No, If yes, describe how.)**

**PROGRESS (Reflection & Future Growth)**

25. **What has been the most effective change or action you have taken in the past months? (Describe a success story or moment of impact.)**
26. **What challenges or barriers have you faced in addressing microaggressions?**
27. **How have faculty or peers responded to your efforts? Have you noticed any shifts in their attitudes or behaviors?**

Date:

**MONITOR (Awareness & Observations)**

28. **How has your awareness of microaggressions evolved over the past months? (Reflect on any new insights, realizations, or knowledge gained.)**
29. **Have you encountered microaggressions in clinical settings or academic discussions? (Describe specific incidents—what happened, and what impact it had.)**
30. **Have you recognized any microaggressive behaviors in yourself that you have worked to change? (Yes/No, If yes, describe the change.)**

**ACT (Personal Strategies & Goals)**

28. **What strategies have you used in the past months to address or mitigate microaggressions?**
29. **Have you set any personal goals if not set 2-3 new personal goals for the next three months on how to aim to eliminate microaggressions.**
30. **Have you started mentoring or guiding others in recognizing and addressing microaggressions? (Yes/No, If yes, describe how.)**

**PROGRESS (Reflection & Future Growth)**

28. **What has been the most effective change or action you have taken in the past months? (Describe a success story or moment of impact.)**
29. **What challenges or barriers have you faced in addressing microaggressions?**
30. **How have faculty or peers responded to your efforts? Have you noticed any shifts in their attitudes or behaviors?**

Date:

**MONITOR (Awareness & Observations)**

31. **How has your awareness of microaggressions evolved over the past months? (Reflect on any new insights, realizations, or knowledge gained.)**
32. **Have you encountered microaggressions in clinical settings or academic discussions? (Describe specific incidents—what happened, and what impact it had.)**
33. **Have you recognized any microaggressive behaviors in yourself that you have worked to change? (Yes/No, If yes, describe the change.)**

**ACT (Personal Strategies & Goals)**

31. **What strategies have you used in the past months to address or mitigate microaggressions?**
32. **Have you set any personal goals if not set 2-3 new personal goals for the next three months on how to aim to eliminate microaggressions.**
33. **Have you started mentoring or guiding others in recognizing and addressing microaggressions? (Yes/No, If yes, describe how.)**

**PROGRESS (Reflection & Future Growth)**

31. **What has been the most effective change or action you have taken in the past months? (Describe a success story or moment of impact.)**
32. **What challenges or barriers have you faced in addressing microaggressions?**
33. **How have faculty or peers responded to your efforts? Have you noticed any shifts in their attitudes or behaviors?**
