# Supplementary material for: Every Word and Action: Using Cinemeducation to Enhance Medical Students’ Awareness of and Upstander Responses to Disability Microaggressions
Source: MedEdPORTAL. 2026 Aug 14;22:11626. doi: 10.15766/mep_2374-8265.11626 (PMC13473049; doi:10.15766/mep_2374-8265.11626)
Supplement: Supplementary file 1 [file mep_2374-8265.11626-s001.zip › J. Confidence Questionnaire.docx]

**Confidence Self-Assessment**

**Note:** Should be used after the 6 months change period. Remove the indicated theme from the items when implementing.

**How confident are you in the following areas?**
Rate **Before** and **After** the session on a scale of **1 to 5**:
1 = Not at all confident
2 = Slightly confident
3 = Moderately confident
4 = Very confident
5 = Extremely confident

| No. | Item | Before Training | After Training |
| --- | --- | --- | --- |
| 1 | Identifying subtle biases when someone makes assumptions about a colleague’s abilities due to a visible or invisible disability (Assuming Incompetence) | ☐ 1 ☐ 2 ☐ 3 ☐ 4 ☐ 5 | ☐ 1 ☐ 2 ☐ 3 ☐ 4 ☐ 5 |
| 2 | Recognizing when language used to "praise" someone with a disability may come across as patronizing or diminishing (Patronizing Language) | ☐ 1 ☐ 2 ☐ 3 ☐ 4 ☐ 5 | ☐ 1 ☐ 2 ☐ 3 ☐ 4 ☐ 5 |
| 3 | Ensuring that all team members, including those with disabilities, are given meaningful roles and responsibilities (Exclusion) | ☐ 1 ☐ 2 ☐ 3 ☐ 4 ☐ 5 | ☐ 1 ☐ 2 ☐ 3 ☐ 4 ☐ 5 |
| 4 | Providing accommodations or adjustments without treating them as privileges or special favors (Offering Rights) | ☐ 1 ☐ 2 ☐ 3 ☐ 4 ☐ 5 | ☐ 1 ☐ 2 ☐ 3 ☐ 4 ☐ 5 |
| 5 | Navigating conversations around disability without using the person’s condition as unrelated inspiration (Overt Inspiration) | ☐ 1 ☐ 2 ☐ 3 ☐ 4 ☐ 5 | ☐ 1 ☐ 2 ☐ 3 ☐ 4 ☐ 5 |
| 6 | Proactively planning for accessible environments without waiting for someone to request adjustments (Accessibility as an Afterthought) | ☐ 1 ☐ 2 ☐ 3 ☐ 4 ☐ 5 | ☐ 1 ☐ 2 ☐ 3 ☐ 4 ☐ 5 |
| 7 | Understanding that disabilities are not always visible and respecting accommodations for invisible conditions (Assuming Disability is Always Visible) | ☐ 1 ☐ 2 ☐ 3 ☐ 4 ☐ 5 | ☐ 1 ☐ 2 ☐ 3 ☐ 4 ☐ 5 |
| 8 | Addressing dismissive comments or minimizing language directed at individuals with disabilities in group settings (Dismissive Comments) | ☐ 1 ☐ 2 ☐ 3 ☐ 4 ☐ 5 | ☐ 1 ☐ 2 ☐ 3 ☐ 4 ☐ 5 |
| 9 | Individualizing care or support for someone with a disability rather than relying on assumptions of what all might need in common. (Overgeneralization) | ☐ 1 ☐ 2 ☐ 3 ☐ 4 ☐ 5 | ☐ 1 ☐ 2 ☐ 3 ☐ 4 ☐ 5 |
| 10 | Responding to expressions of sympathy that might feel uncomfortable to individuals with disabilities (Excessive Sympathy) | ☐ 1 ☐ 2 ☐ 3 ☐ 4 ☐ 5 | ☐ 1 ☐ 2 ☐ 3 ☐ 4 ☐ 5 |
| 11 | Respecting personal boundaries by avoiding overly personal questions about someone’s disability (Intrusive Questions) | ☐ 1 ☐ 2 ☐ 3 ☐ 4 ☐ 5 | ☐ 1 ☐ 2 ☐ 3 ☐ 4 ☐ 5 |
| 12 | Ensuring patient-centered interactions by addressing individuals directly and not speaking about them to others. (Speaking Over) | ☐ 1 ☐ 2 ☐ 3 ☐ 4 ☐ 5 | ☐ 1 ☐ 2 ☐ 3 ☐ 4 ☐ 5 |
