# Supplementary material for: Every Word and Action: Using Cinemeducation to Enhance Medical Students’ Awareness of and Upstander Responses to Disability Microaggressions
Source: MedEdPORTAL. 2026 Aug 14;22:11626. doi: 10.15766/mep_2374-8265.11626 (PMC13473049; doi:10.15766/mep_2374-8265.11626)
Supplement: Supplementary file 1 [file mep_2374-8265.11626-s001.zip › K. Semi-Structured Interview Prompts.docx]

**Semi Structured Interview Prompts to Build on**

*Note: Should be used during follow-up interviews after the session.*

1. Can you describe your initial thoughts when you first heard about the cinemeducation session?
2. How did the experience of watching curated film clips differ from traditional lectures or textbooks?

3. Were there specific scenes or themes from the film clips that stood out to you or emotionally affected you? Why?
4. How did the session help you personally connect with the challenges faced by persons with disabilities?
5. What thoughts or realizations did the reflection sheet prompt for you during the session?

6. Before the session, how familiar were you with the concept of microaggressions against persons with disabilities?
7. Has your ability to recognize subtle or unintentional discrimination improved? Can you share an example?
8. Did you identify any personal biases or assumptions during or after the session?

9. How did you feel about the small group discussions? Did they encourage you to share openly?
10. Did any peer perspectives shift your understanding of disability inclusion?

11. Can you share an example from the last 6 months where you noticed or responded to a microaggression?
12. Did you act as an upstander or help educate a peer about inclusive behavior?
13. How did maintaining the MAP diary influence your awareness and actions?

14. How has your confidence changed in addressing microaggressions or standing up for disability rights?
15. What specific inclusive practices have you started applying in academic or clinical interactions?

16. What improvements would you recommend for future cinemeducation sessions?
17. Should this approach be more widely included in the medical curriculum? Why or why not?

18. What is one lasting lesson or takeaway from this experience that you will carry into your medical career?
19. Is there anything else you’d like to share about your personal transformation or reflections?
