# Supplementary material for: Every Word and Action: Using Cinemeducation to Enhance Medical Students’ Awareness of and Upstander Responses to Disability Microaggressions
Source: MedEdPORTAL. 2026 Aug 14;22:11626. doi: 10.15766/mep_2374-8265.11626 (PMC13473049; doi:10.15766/mep_2374-8265.11626)
Supplement: Supplementary file 1 [file mep_2374-8265.11626-s001.zip › B. Microaggression Themes.docx]

**Microaggressions Themes & Description**

Note: Should be used before the session during planning and referred to during facilitation.

| Theme | Description |
| --- | --- |
| 1. Assuming Incompetence | Believing that individuals with disabilities lack the ability, intelligence, or capability to succeed, based on biased assumptions rather than actual evidence. |
| 2. Patronizing Language | Using overly simplistic, condescending, or infantilizing language that undermines the dignity and autonomy of the individual. |
| 3. Exclusion | Preventing or limiting the full participation of individuals with disabilities in environments due to physical, structural, or social barriers. |
| 4. Offering Rights | Treating basic rights such as access and independence as optional or as favors, rather than recognizing them as entitlements. |
| 5. Overt Inspiration | Excessively praising individuals with disabilities for routine actions, implying their worth is tied to overcoming perceived limitations. |
| 6. Accessibility as an Afterthought | Considering accessibility only after challenges arise, rather than embedding it into initial planning and design. |
| 7. Assuming Disability is Always Visible | Believing that disabilities are only physical and outwardly noticeable, disregarding hidden or invisible conditions. |
| 8. Dismissive Comments | Making remarks that minimize, invalidate, or overlook the lived experiences and needs of individuals with disabilities. |
| 9. Overgeneralization | Applying a single narrative or stereotype to all individuals with disabilities, ignoring diversity within the disability community. |
| 10. Excessive Sympathy | Expressing disproportionate pity that portrays individuals with disabilities as inherently helpless or suffering. |
| 11. Intrusive Questions | Asking inappropriate or overly personal questions about someone’s disability, violating their boundaries or privacy. |
| 12. Speaking Over | Discussing or making decisions about individuals with disabilities without including their voice or input in the process. |
