# Supplementary material for: Every Word and Action: Using Cinemeducation to Enhance Medical Students’ Awareness of and Upstander Responses to Disability Microaggressions
Source: MedEdPORTAL. 2026 Aug 14;22:11626. doi: 10.15766/mep_2374-8265.11626 (PMC13473049; doi:10.15766/mep_2374-8265.11626)
Supplement: Supplementary file 1 [file mep_2374-8265.11626-s001.zip › C. Movie Citations and Time Stamps.docx]

**Appendix C**

**Movie Citations & Time Stamps**

**Note:** For each clip, the source film, timestamp, scene description, explanation of how the scene relates to the identified theme, availability of the source, and guidance for selecting alternative clips have been provided.

The educational value of a clip lies primarily in the interaction, behaviour, and theme depicted rather than the specific film itself. Educators may therefore use alternative clips that demonstrate similar situations and align with the same learning objectives while remaining culturally, linguistically, and contextually appropriate for their learners.

For non-English clips, scene descriptions have been included to facilitate implementation in diverse educational settings.

**1. THEME 1: ASSUMING INCOMPETENCE**

**Film Title: *Forrest Gump*
Scene Title: *“Run, Forrest, Run!” Scene*
Director: Robert Zemeckis
Year: 1994**

**Scene Time Stamp: 15.42 - 17.40**

**Scene Description:** In this scene, young Forrest is walking home wearing leg braces when a group of boys begin throwing stones at him. Jenny, his friend tells Forrest ***“Run, Forrest, Run!”***, and the boys chase him on their bicycles. As Forrest runs away, his leg braces break apart and he continues running while the boys are unable to catch him.

**Sources where this movie is available:** Jio Hotstar, Amazon Prime, YouTube, Google PlayMovies

**How this related to the theme:** This scene demonstrates how people often make assumptions about the abilities of persons with disabilities without truly knowing what they are capable of. The boys automatically view Forrest as weak and unable to protect himself because of his physical disability. Their actions are driven by the belief that he is less capable than others. The scene highlights how assumptions about incompetence can limit opportunities, influence behaviour towards individuals with disabilities, and reinforce unfair stereotypes.

**What to look for in alternate clips:**

- A person with a disability is presumed incapable without evidence.
- Others make decisions on their behalf without consultation.
- Opportunities are denied because of assumptions about ability.
- Communication is directed to others instead of the individual.
- The scene clearly demonstrates underestimation of competence rather than actual inability.

**Citation:** *Forrest Gump. Directed by Robert Zemeckis. The Steve Tisch Company, Wendy Finerman Productions, Paramount Pictures; 1994.*

**2. THEME 2: PATRONIZING LANGUAGE**

**Film Title: *Daredevil*
Director: Mark Steven Johnson
Year: 2003**

**Scene Time Stamp: 31.00 - 34.08**

**Scene Description:** Matt Murdock, a blind lawyer, is at a restaurant with his friend Foggy Nelson and a woman. When the woman offers Matt the sauce in front of him, Foggy sarcastically overexplains that Matt is blind and jokes about his limitations. When the woman refuses to share her name, Foggy mockingly says, “Some people have no compassion for the handicapped,” using Matt’s blindness to make a point that isn’t actually empathetic.

**Sources where this movie is available:** Jio Hotstar, YouTube

**How this related to the theme:** This scene illustrates patronizing language and attitudes towards persons with disabilities. Although the comments may appear helpful on the surface, they are based on assumptions that Matt is less capable and requires special treatment. The repeated emphasis on his blindness, unnecessary assistance, and sarcastic references to compassion undermine his independence and dignity. The scene provides an opportunity to discuss how seemingly well-intentioned language can become condescending and contribute to disability-related microaggressions.

**What to look for in alternate clips:**

- Use of childlike, oversimplified, or condescending language.
- Excessive praise for routine activities.
- Speaking in a tone that diminishes autonomy or maturity.
- Interactions that appear well-intentioned but undermine dignity.
- Clear contrast between respectful and patronizing communication.

**Citation:** *Daredevil. Directed by Mark Steven Johnson. Marvel Enterprises, New Regency, Horseshoe Bay Productions, Twentieth Century Fox; 2003.*

**3. THEME 3: EXCLUSION**

**Film Title: *Ratsasan***

***Langauge: Tamil*
Director: Ram Kumar
Year: 2018**

**Scene Time Stamp: 1.58.28 - 2.01.18**

**Scene Time Stamp: 31.00 - 34.08**

**Scene Description:** In this flashback scene, Christopher arrives at school during lunchtime. As he approaches a table, the other students move away and avoid sitting with him. He is left alone while the other children keep their distance. A girl named Sophie notices this and invites Christopher to sit with her. The two of them then share lunch together. Because of this repeated exclusion, later in the movie he becomes an anti-social personality.

**Sources where this movie is available:** Jio Hotstar, Sun NXT app (with English subtitles)

**How this related to the theme:** This scene illustrates exclusion through the way Christopher is socially isolated by his peers because of his appearance resulting from Progeria. While the other students choose not to interact with him, Sophie treats him as an equal and includes him in a normal social activity. The contrast highlights how exclusion can occur in everyday situations and how such acts damage the person and leads to harmful and revengeful personalities.

**What to look for in alternate clips:**

- A person with a disability is physically present but socially excluded.
- Exclusion from group activities, discussions, or decision-making.
- Others ignore, overlook, or bypass the individual.
- The exclusion is subtle rather than overt discrimination.
- A scene that shows emotional impact of being left out.

**Citation:** *Ratsasan. Directed by Ram Kumar. Axess Film Factory; 2018*

**4. THEME 4: OFFERING RIGHTS**

**Film Title: *The Intouchables*
Director: Olivier Nakache and Éric Toledano
Year: 2011**

**Scene Time Stamp: 22.54 - 29.50**

**Scene Time Stamp: 31.00 - 34.08**

**Scene Description:** In this scene, Philippe, a man with quadriplegia, is being assisted during mealtime. One of the caregivers feeds him and helps him with his daily needs. Throughout the interaction, the caregiver displays impatience through his tone, facial expressions, and manner of speaking. Philippe observes these reactions while the caregiver continues assisting him.

**Sources where this movie is available:** Amazon Prime, YouTube, Google PlayMovies

**How this related to the theme:** This scene illustrates how support and assistance can sometimes be provided in a way that suggests they are favors rather than basic rights. The caregiver's attitude and behaviour convey a sense of superiority, implying that Philippe should be grateful for receiving assistance with his daily needs. The scene encourages discussion about dignity, respect, and the importance of viewing access, support, and care as rights rather than acts of generosity.

**What to look for in alternate clips:**

- Basic rights or accommodations are portrayed as favors.
- Inclusion is presented as generosity rather than entitlement.
- A character is expected to be grateful for equal treatment.
- Access is framed as a special privilege.
- A scene that shows some interaction highlighting unequal power dynamics.

**Citation:** *The Intouchables. Directed by Éric Toledano and Olivier Nakache. Gaumont; Quad Films; TF1 Films Production; Ten Films; Chaocorp; Canal+; CinéCinéma; 2011.*

**5. THEME 5: OVERT INSPIRATION**

**Film Title: *Don’t Look Down on Me* (Short Film)
Creator: Kat Pemberton (Fixers Project, 2017)**

**Scene Time Stamp: 0.15 - 4.43 (full video)**

**Scene Description:** In this short film, a young woman with a disability speaks directly about her experiences and the way people respond to her in everyday life. She describes situations where others praise her for routine activities and achievements. Through a series of examples, she explains the comments she receives and how these interactions make her feel.

**Sources where this movie is available:** YouTube

**How this related to the theme:** This clip illustrates overt inspiration by showing how ordinary activities performed by a person with a disability are sometimes viewed as extraordinary. The repeated praise and admiration are directed towards the disability rather than the individual's actual achievements. The scene provides an opportunity to discuss how such responses, although often well-intentioned, can be uncomfortable and may reduce a person to their disability rather than recognising them as an individual.

**What to look for in alternate clips:**

- A person with a disability is portrayed primarily as an inspiration.
- Routine activities are celebrated as extraordinary solely because of disability.
- Disability becomes the focus rather than the person's individuality.
- The portrayal evokes admiration without meaningful understanding.
- A scene that can distinguish inspiration from stereotyping.

**Citation:** *Don’t Look Down on Me. Created by Kat Pemberton; Fixers Project; 2017.*

**6. THEME 6: ACCESSIBILITY AS AN AFTERTHOUGHT**

**Film Title: *Thanga Meengal***

***Language: (Tamil)*
Director: Ram
Year: 2013**

**Scene Time Stamp: 12.52 - 15.11 & 49.28 - 51.03**

**Scene Description:** In these scenes, a schoolgirl names Chellamma struggles academically and is frequently criticized for her performance in school. She is compared with other students and finds it difficult to meet the expectations placed on her. Later, her father meets the school authorities (principal and teacher) to discuss her difficulties and seeks an explanation for her challenges. Only after this discussion do the school staff begin to examine her situation more closely and consider her needs.

**Sources where this movie is available:** Sun NXT (with English subtitles), Airtel Xstream Play, YouTube, Amazon Prime (with English subtitles)

**How this related to the theme:** This scene demonstrates accessibility as an afterthought. The student's difficulties are initially viewed as poor performance rather than a need for additional support. Consideration of her learning needs occurs only after concerns are raised by her father. The clip encourages discussion about the importance of identifying barriers early and creating supportive environments that allow all students to participate and succeed on an equal basis.

**What to look for in alternate clips:**

- Accessibility needs are overlooked during planning.
- Barriers become apparent only after a problem occurs.
- A person is unintentionally excluded because accessibility was not considered.
- Environmental or systemic obstacles are highlighted.
- A scene that shows how focus is on impairment itself than preventable barriers

**Citation:** *Thanga Meengal. Directed by Ram. Photon Kathaas, JSK Film Corporation; 2013.*

**7. THEME 7: ASSUMING DIABILITY IS ALWAYS VISIBLE**

**Film Title: *Silver Linings Playbook*
Director: David O. Russell
Year: 2012**

**Scene Time Stamp:  26.48 - 29.25**

**Scene Description:** In this scene, Pat and Tiffany are having dinner and talking about their experiences with mental health conditions. During the conversation, Tiffany speaks about her medications and the challenges she faces. As the discussion continues, the reactions of others at the table make her increasingly uncomfortable. Tiffany becomes upset and decides to leave rather than continue the conversation.

**Sources where this movie is available:** Amazon Prime, YouTube, Google PlayMovies

**How this related to the theme:** This scene highlights the misconception that disability is always visible. Both characters are living with mental health conditions, yet their difficulties are not immediately apparent to others. The reactions of those around Tiffany demonstrate how invisible disabilities are often misunderstood, dismissed, or judged because there are no obvious physical signs. The clip encourages discussion about the importance of recognising psychological and psychosocial disabilities alongside visible forms of disability.

**What to look for in alternate clips:**

- A person's disability or accommodation needs are questioned.
- Others doubt the legitimacy of an invisible disability.
- Symptoms or limitations are dismissed because they are not visible.
- Assumptions are made based solely on appearance.
- A scene that demonstrates misconceptions about hidden disabilities.

**Citation:** *Silver Linings Playbook. Directed by David O. Russell. Mirage Enterprises; The Weinstein Company; 2012.*

**8. THEME 8: DISMISSIVE COMMENTS**

**Film Title: *The Intouchables*
Directors: Olivier Nakache and Éric Toledano
Year: 2011**

**Scene Time Stamp: 32.32 - 34.30**

**Scene Description:** In this scene, Philippe expresses his interest in art and discusses purchasing an expensive painting. His caregiver, Driss, disagrees with the decision and repeatedly questions the value of the artwork. As the conversation continues, Driss repeatedly mocks Philippe by saying “No feet, no candy” whenever Philippe asks for candy. Philippe's requests and opinions are met with teasing and dismissive remarks throughout the interaction.

**Sources where this movie is available:** Amazon Prime, YouTube, Google PlayMovies

**How this related to the theme:** This scene illustrates dismissive comments through the repeated mocking and trivialisation of Philippe's views and requests. Rather than engaging respectfully with Philippe, Driss minimizes his preferences and uses humour in a way that undermines his perspective. The clip provides an opportunity to discuss how dismissive remarks, even when intended as jokes, can affect dignity, respect, and interpersonal relationships for persons with disabilities.

**What to look for in alternate clips:**

- Concerns raised by a person with a disability are minimized.
- Statements such as "it's not a big deal" or similar invalidation.
- Emotional experiences are ignored or trivialized.
- The individual feels unheard or unsupported.
- A scene where impact of dismissive communication is evident.

**Citation:** *The Intouchables. Directed by Olivier Nakache and Éric Toledano. Gaumont; Quad Films; TF1 Films Production; Ten Films; Chaocorp; Canal+; CinéCinéma; 2011.*

**9. THEME 9: OVER GENERALIZATION**

**Series Title: *The Good Doctor*
Creator: David Shore**

**Scene Time Stamp: Season 1, episode 7 : 3.00 - 4.00**

**Scene Description:** In this scene, a young patient arrives at the hospital appearing distressed and agitated. Several members of the healthcare team assume he is experiencing a psychotic episode and discuss managing him accordingly. Dr. Shaun Murphy observes the patient and interacts with him directly. After spending time with him, Shaun tells the team, **"He is not psychotic. He's autistic."** Shaun then adjusts his communication and approach, helping the patient become calmer and more cooperative.

**Sources where this movie is available:** Netflix, YouTube

**How this related to the theme:** This scene illustrates overgeneralization by showing how healthcare professionals make assumptions about a patient based on limited observations. The patient's behaviour is quickly attributed to psychosis without a proper understanding of his condition. Shaun recognizes that the patient is autistic and requires a different approach. The clip highlights the dangers of applying broad stereotypes to individuals and reinforces the importance of understanding each person's unique needs and experiences rather than making assumptions based on labels or behaviours alone.

**What to look for in alternate clips:**

- Assumptions about one person are extended to an entire disability group.
- Stereotypes replace individualized understanding.
- A disability label becomes the basis for broad conclusions.
- Unique abilities, preferences, or experiences are overlooked.
- A scene that highlights the diversity within disability communities.

**Citation:** *“The Good Doctor.” Produced by Shore Z Productions, 3AD, EnterMedia, and ABC Signature (as ABC Studios in Season 1); created by David Shore. Season 1, Episode 7; 2017.*

**10. THEME 10: EXCESSIVE SYMPATHY**

**Film Title: *Soul Surfer*
Director: Sean McNamara
Year: 2011**

**Scene Time Stamp: 43.41 - 45.50**

**Scene Description:** In this scene, Bethany returns to meet her friends after losing her arm in a shark attack. During the interaction, her friends express concern and sympathy for what she has experienced. They speak about praying for her and offer words of comfort and encouragement. Bethany listens to these conversations while continuing to cope with the emotional impact of her injury.

**Sources where this movie is available:** YouTube, Amazon Prime

**How this related to the theme:** This scene illustrates excessive sympathy by showing how interactions can become centred on a person's disability or loss. While the concern expressed by Bethany's friends is genuine, much of the conversation focuses on what has happened to her rather than on her as an individual. The clip provides an opportunity to discuss the difference between empathy and excessive sympathy, and how well-intentioned responses can sometimes make individuals feel defined by their condition rather than supported in moving forward.

**What to look for in alternate clips:**

- Interactions are dominated by pity rather than respect.
- The person is treated as helpless or tragic.
- Sympathy overshadows autonomy and strengths.
- Others focus excessively on loss or suffering.
- The distinction between empathy and pity can be discussed.

**Citation:** *Soul Surfer. Directed by Sean McNamara. Enticing Entertainment; Island Film Group; Affirm Films; Brookwell McNamara Entertainment; Life’s a Beach Entertainment; Mandalay Vision; 2011.*

**11. THEME 11: INTRUSIVE QUESTIONS**

**Film Title: *Wonder*
Director: Stephen Chbosky
Year: 2017**

**Scene Time Stamp: 07.13 - 10.18**

**Scene Description:** In this scene, Auggie meets his classmates for the first time at school. During the conversation, one student asks him whether he knows what an eraser is and then begins asking questions about his facial appearance. Although another student attempts to stop the questioning, she also explains his appearance by saying that he was born that way. Throughout the interaction, Auggie listens to the comments and responds to the questions while trying to remain composed.

**Sources where this movie is available:** Amazon Prime, YouTube, Google PlayMovies

**How this related to the theme:** This scene illustrates intrusive questions by showing how curiosity can sometimes override respect for personal boundaries. Auggie is repeatedly asked questions about his appearance in a public setting without consideration of how uncomfortable these questions may be for him. While the students may not intend to be hurtful, the interaction places Auggie in a position where he is expected to explain and justify his difference. The clip provides an opportunity to discuss privacy, boundaries, and respectful communication when interacting with persons with disabilities or visible differences.

**What to look for in alternate clips:**

- Personal questions are asked without invitation/ in a way that makes the person awkward in public.
- Curiosity overrides respect for privacy.
- The person is pressured to explain their condition.
- Boundaries are ignored or crossed.
- A scene that shows how interaction creates discomfort or loss of agency.

**Citation:** *Wonder. Directed by Stephen Chbosky. Lionsgate; Mandeville Films; Participant Media; Walden Media; TIK Films; 2017*

**12. THEME 12: SPEAKING OVER**

**Produced by: South Australian Office of the Commissioner for Equal Opportunity
Year: 2021
Type: Workplace Training Video**

**Scene Time Stamp: 0.09 - 3.46 (full video)**

**Scene Description:** In this workplace training video, Roger requests a suitable chair and workstation adjustment after sustaining a back injury. While discussing the request, the manager and secretary speak about Roger and his situation without directly involving him in the conversation. Later, when the secretary meets Roger, she repeatedly focuses on his condition and suggests that he should rest rather than discussing the adjustments he has requested. As Roger attempts to explain his needs and concerns, the conversation continues to be directed by others rather than by Roger himself.

**Sources where this movie is available:** YouTube

**How this related to the theme:** This scene illustrates speaking over, where decisions and discussions concerning a person are conducted without meaningfully involving them. Roger's needs and preferences are repeatedly overshadowed by the assumptions and opinions of others. Rather than listening to his perspective, the manager and secretary make decisions on his behalf and focus on what they believe is best for him. The clip highlights how speaking over a person can reduce autonomy, limit participation in decision-making, and create a sense of powerlessness.

**What to look for in alternate clips:**

- Others speak on behalf of the person without permission.
- Questions are directed to caregivers, friends, or family members without discussing with the person involved.
- The individual is not given an opportunity to respond.
- Communication bypasses the person concerned.
- A scene that demonstrates loss of voice, agency, or participation.

**Citation:** *Workplace training video. Produced by South Australian Office of the Commissioner for Equal Opportunity; 2021.*
