# Supplementary material for: Every Word and Action: Using Cinemeducation to Enhance Medical Students’ Awareness of and Upstander Responses to Disability Microaggressions
Source: MedEdPORTAL. 2026 Aug 14;22:11626. doi: 10.15766/mep_2374-8265.11626 (PMC13473049; doi:10.15766/mep_2374-8265.11626)
Supplement: Supplementary file 1 [file mep_2374-8265.11626-s001.zip › D. Pre- and Posttest.docx]

**PRE / POST TEST**

**(**Should be used before the session and immediately after the session)

**1. During a simulation exercise, a student with a disability is asked to monitor rather than perform a procedure, with the assumption that it may be "too much to handle."
What hidden bias is present in this decision?**

**2. After a routine clinical task, a colleague comments to a peer with a disability, “You’re doing great, despite everything!” What impact does this language most likely have on the peer’s experience?**

**3. In a team meeting, a student with an invisible disability is left out of task assignments under the assumption that they might need additional rest. Which aspect of this decision highlights unintentional exclusion?**

**4. A supervisor remarks that they’re “making an exception” by allowing a colleague with specific needs a more flexible schedule, although it’s a formal accommodation. How does this comment affect the perceived status of accommodations?**

**5. A team lead remarks that an individual with a disability is “an inspiration just for being here,” even though they’re excelling in the same way as everyone else. What issue is present in this comment?**

**6. A conference event is initially scheduled in a space without wheelchair access, with alternate arrangements made only after a participant raises concerns. What core issue does this reveal about accessibility planning?**

**7. A peer expresses disbelief when a colleague requests a reduced workload due to a non-visible health condition, commenting, “But you don’t look like you need it.” What assumption does this response reflect?**

**8. A patient shares challenges related to their mobility limitations, and a healthcare provider responds, “That’s just a part of aging.” Which aspect of this response could be considered dismissive?**

**9. A resident assumes that every patient with a specific disability shares the same needs, applying the same approach across all cases. What bias is reflected by this assumption?**

**10. During a patient interaction, a healthcare provider frequently expresses pity, saying, “I can’t imagine how hard this must be for you,” even when the patient discusses routine activities. Which impact might this excessive sympathy have on the patient?**

**11. During rounds, a healthcare provider asks a patient with a disability about their life outside of work in detail, even though it’s unrelated to the patient’s current health concern. What is problematic about this approach?**

**12. During a consult, a healthcare provider directs questions about the patient’s condition to their accompanying family member instead of addressing the patient directly. What negative impact does this interaction primarily have?**

**13. A medical student notices that their peers frequently make comments about a classmate’s accommodation needs, emphasizing they receive "special treatment." What form of interaction is this an example of?**

**14. A healthcare provider experiences repeated instances of others making assumptions about their abilities due to a disability, feeling a growing sense of isolation and frustration. What impact can frequent form of this behaviour have on an individual’s professional experience?**

**15. A patient is assumed to require assistance for daily activities based solely on the presence of a physical aid, despite expressing confidence in their independence. What impact might this assumption have on the patient’s autonomy?**

**Instructions for Scoring**

The following scoring guidance applies to the 15 open-ended scenarios included in the pre- and post-test. **Responses should be evaluated based on the learner’s understanding of the situation presented, their ability to explain the concerns raised within the interaction, and the appropriateness of the response proposed within the given context.**

• A score of 0 may be assigned when the response demonstrates limited understanding of the situation or includes an inappropriate interpretation or response.

• A score of 1 may be assigned when the learner demonstrates some understanding of the concerns raised but provides a partially developed, incomplete, or only partly appropriate response.

• A score of 2 should be assigned when the learner demonstrates a clear understanding of the situation, thoughtfully explains the concerns involved, and provides an appropriate, contextually relevant, and respectful response.

Each item carries a maximum of 2 marks, and total scores are calculated by summing across all 15 items. Responses may be scored independently by two faculty members, with discrepancies resolved through discussion to promote scoring consistency.

**Key for Assessors**

**1. For full credit, the student should:**

- Explain that the student is judged based on their disability instead of their actual ability.
- Mention that everyone should be given a fair chance to participate.
- Recognize that this can reduce learning opportunities.

**2. For full credit, the student should:**

- Explain that the comment may sound patronizing, even if it was meant as praise.
- Mention that it focuses on the disability instead of the achievement.
- Recognize that it may make the person feel uncomfortable or underestimated.

**3. For full credit, the student should:**

- Explain that the student was left out based on assumptions.
- Mention that no one asked about the student's preferences or abilities.
- Recognize that everyone should have equal opportunities.

**4. For full credit, the student should:**

- Explain that accommodations are made to seem like special favors.
- Mention that accommodations are a right, not a privilege.
- Recognize that this can increase stigma.

**5. For full credit, the student should:**

- Explain that the person's disability is being highlighted instead of their achievement.
- Mention that the comment may feel patronizing.
- Recognize that achievements should be appreciated without focusing on disability.

**6. For full credit, the student should:**

- Explain that accessibility was considered too late.
- Mention that accessibility should be planned from the beginning.
- Recognize that everyone should be able to participate without asking for changes.

**7. For full credit, the student should:**

- Explain that not all disabilities are visible.
- Mention that appearance should not be used to judge someone's needs.
- Recognize the importance of trusting the person's experience.

**8. For full credit, the student should:**

- Explain that the patient's concerns are being minimized.
- Mention that the provider did not acknowledge the patient's experience.
- Recognize that the concerns should be explored respectfully.

**9. For full credit, the student should:**

- Explain that not all people with the same disability have the same needs.
- Mention that each person should be treated as an individual.
- Recognize that assumptions should be avoided.

**10. For full credit, the student should:**

- Explain that the patient may feel pitied instead of respected.
- Mention that excessive sympathy can affect dignity.
- Recognize that patients should be treated with respect and empathy.

**11. For full credit, the student should:**

- Explain that the questions are unrelated to the patient's care.
- Mention that they may invade the patient's privacy.
- Recognize the importance of respecting personal boundaries.

**12. For full credit, the student should:**

- Explain that the patient is not being included in the conversation.
- Mention that this reduces the patient's independence and dignity.
- Recognize that healthcare providers should speak to the patient first whenever possible.

**13. For full credit, the student should:**

- Explain that this is a disability-related microaggression.
- Mention that calling accommodations "special treatment" can create stigma and sense of discrimination.
- Recognize that accommodations beforehand help provide equal opportunities.

**14. For full credit, the student should:**

- Explain that repeated assumptions can reduce confidence.
- Mention that they may lead to frustration, stress, or feeling excluded.
- Recognize that they can affect a person's sense of belonging at work.

**15. For full credit, the student should:**

- Explain that assuming someone needs help can reduce their independence.
- Mention that the person's own choices and abilities should be respected.
- Recognize that support should be offered only after asking what the person prefers.
