# Supplementary material for: Every Word and Action: Using Cinemeducation to Enhance Medical Students’ Awareness of and Upstander Responses to Disability Microaggressions
Source: MedEdPORTAL. 2026 Aug 14;22:11626. doi: 10.15766/mep_2374-8265.11626 (PMC13473049; doi:10.15766/mep_2374-8265.11626)
Supplement: Supplementary file 1 [file mep_2374-8265.11626-s001.zip › F. Points for Discussion.docx]

**Points of Discussion**

**For Facilitators**

Note: Should be used during the session while facilitating discussions.

**1. Assuming Incompetence**

**Film Title:** *Forrest Gump*
**Director:** Robert Zemeckis
**Year:** 1994
**Language:** English
**Scene Description:** Forrest is chased by bullies who assume his disability will prevent him from escaping. Even his friend urges him to run rather than stand up for himself. When he runs freely for the first time, he breaks the assumption of helplessness.
**Theme Alignment:** The clip reflects the assumption that visible disability equates to inability. Forrest’s unexpected ability to run challenges prejudiced beliefs about competence.

**Points for Discussion:**

- What assumptions are made about Forrest’s abilities based on his disability?
- How does Forrest’s ability to run challenge stereotypes and expectations?
- How might Forrest have felt before and after being chased?
- Have you encountered or witnessed similar assumptions in clinical or academic environments?
- What can we learn about how society underestimates people with disabilities?

**2. Patronizing Language**

**Film Title:** *Daredevil*
**Director:** Mark Steven Johnson
**Year:** 2003
**Language:** English
**Scene Description:** Matt Murdock, a blind man, is at dinner when his friend sarcastically highlights his blindness in front of a woman. The friend comments that people lack compassion for “the handicapped” in a patronizing tone.
**Theme Alignment:** The clip shows how sarcastic language and exaggerated “sympathy” can feel demeaning, even if presented as humor or concern. It undermines the individual’s autonomy.

**Points for Discussion:**

- What language and gestures make the friend's behavior seem condescending?
- Does using a kind tone excuse patronizing content? Why or why not?
- How does sarcasm about disability reflect deeper prejudice?
- What is the impact on the self-esteem and dignity of the person being addressed?
- How can we ensure we speak respectfully while still offering support?

**3. Exclusion**

**Film Title:** *Ratsasan*
**Director:** Ram Kumar
**Year:** 2018
**Language:** Tamil
**Scene Description:** A boy with unusual facial features is bullied, mocked, and rejected by his peers. The resulting trauma and exclusion contribute to psychological breakdown and later violent behavior.
**Theme Alignment:** The clip captures how exclusion based on appearance leads to long-term emotional damage and reinforces social rejection.

**Points for Discussion:**

- What triggers the exclusion of the boy from classroom participation?
- How do repeated acts of exclusion affect mental health and social behavior?
- What forms of subtle exclusion exist in schools or clinics?
- How do visual or behavioral differences become the basis of bias?
- What role should educators and peers play in preventing social isolation?

**4. Offering Rights**

**Film Title:** *The Intouchables*
**Directors:** Olivier Nakache and Éric Toledano
**Year:** 2011
**Language:** English
**Scene Description:** Philippe, a wealthy man with quadriplegia, is fed by a formal caregiver who behaves with visible discomfort and condescension. The caregiver acts as though offering basic help is a burden, showing frustration through facial expressions.
**Theme Alignment:** This scene powerfully depicts how basic human rights such as dignity and autonomy are sometimes presented as favors. The imbalance between caregiver and care recipient becomes a subtle but impactful form of microaggression.

**Points for Discussion:**

- What makes the caregiver’s actions feel more like offering a favor than respectful care?
- How might Philippe feel during this moment of being fed?
- What is the difference between providing support and controlling someone's routine?
- In what ways does the caregiver’s attitude affect the dignity of the recipient?
- How can caregivers promote respect while performing essential tasks?

**5. Overt Inspiration**

**Short Film Title:** *Don’t Look Down on Me*
**Creator:** Kat Pemberton (Fixers UK Project)
**Year:** 2017
**Language:** English
**Scene Description:** Kat, a wheelchair user, shares how people often praise her for simply performing normal tasks, like going out or posting online.
**Theme Alignment:** The film critiques how excessive praise can reduce a person’s identity to their disability, turning everyday actions into spectacles of “inspiration.”

**Points for Discussion:**

- Why might excessive praise feel uncomfortable for people with disabilities?
- Is being called an “inspiration” always meant as a compliment?
- When does admiration cross the line into objectification?
- How do media and society contribute to ‘inspiration porn’ narratives?
- What are ways to genuinely acknowledge someone’s strength without stereotyping?

**6. Accessibility as an Afterthought**

**Film Title:** *Thanga Meengal*
**Director:** Ram
**Year:** 2013
**Language:** Tamil
**Scene Description:** A schoolgirl with a learning disability is excluded from activities and scolded unfairly. Only after her father raises concerns does the school begin to reflect on their treatment.
**Theme Alignment:** The clip highlights how systems fail to support inclusivity unless prompted by external confrontation, making accessibility a reactive effort.

**Points for Discussion:**

- How does the school respond to the girl's learning challenges?
- What emotional toll does exclusion take on a student?
- Why is early recognition and support critical in inclusive education?
- How can institutional delays in accessibility be prevented?
- How do family voices help push for systemic change?

**7. Assuming Disability is Always Visible**

**Film Title:** *Silver Linings Playbook*
**Director:** David O. Russell
**Year:** 2012
**Language:** English
**Scene Description:** Tiffany speaks openly about her mental health and medications, but others respond with discomfort and misunderstanding.
**Theme Alignment:** The clip demonstrates how invisible disabilities like mental illness are overlooked, misinterpreted, or dismissed as poor behavior.

**Points for Discussion:**

- What assumptions are made about what disability “should” look like?
- How does the girl’s discomfort reflect the burden of invisible disabilities?
- Why is there a need for empathy beyond what can be seen?
- What does the dinner scene reveal about public understanding of mental health?
- How can medical education address the nuances of psychosocial disabilities?

**8. Dismissive Comments**

**Film Title:** *The Intouchables*
**Directors:** Olivier Nakache and Éric Toledano
**Year:** 2011
**Language:** English
**Scene Description:** Philippe, a man with quadriplegia, expresses interest in abstract painting. His caregiver mocks the art and makes dismissive jokes.
**Theme Alignment:** The clip shows how a disabled person’s preferences or interests can be minimized through sarcasm, making their autonomy feel unvalued.

**Points for Discussion:**

- What does the repeated mocking line “No feet, no candy” imply about the caregiver’s respect?
- How do offhand comments undermine a person’s dignity and autonomy?
- What power imbalance is shown in the way the assistant communicates?
- How might such language affect the emotional well-being of the person being cared for?
- How can caregivers and colleagues become more conscious of dismissive language?

**9. Overgeneralization**

**Series Title:** *The Good Doctor*
**Creator:** David Shore
**Language:** English
**Clip Source:** Public YouTube scene – emergency room episode
**Scene Description:** A boy in distress is presumed to be psychotic. Dr. Murphy, an autistic doctor, correctly identifies the child as autistic, not violent.
**Theme Alignment:** The scene critiques how one-size-fits-all thinking leads to incorrect, harmful assumptions about behavior linked to disability.

**Points for Discussion:**

- Why is the young patient immediately labeled psychotic?
- How does Dr. Murphy’s insight challenge the bias of other healthcare staff?
- What risks come with quick judgments in clinical settings?
- How are neurodivergent individuals often misunderstood?
- What can healthcare teams do to pause and assess without stereotyping?

**10. Excessive Sympathy**

**Film Title:** *Soul Surfer*
**Director:** Sean McNamara
**Year:** 2011
**Language:** English
**Scene Description:** Bethany, after losing her arm, is overwhelmed by people showing pity and offering unsolicited support, despite her efforts to move forward.
**Theme Alignment:** The clip illustrates how excessive sympathy isolates rather than empowers, focusing on disability instead of strength.

**Points for Discussion:**

- Why can constant sympathy feel overwhelming or uncomfortable?
- How do well-meaning comments sometimes intensify grief or frustration?
- What does the character’s internal struggle reveal about visible versus hidden emotional pain?
- How can empathy be offered without making someone feel pitied?
- How can we affirm resilience while respecting boundaries?

**11. Intrusive Questions**

**Film Title:** *Wonder*
**Director:** Stephen Chbosky
**Year:** 2017
**Language:** English
**Scene Description:** Auggie is asked invasive questions about his facial features by a classmate. Even an attempt to defend him unintentionally emphasizes his difference.
**Theme Alignment:** The clip highlights how curiosity, when unchecked, can violate boundaries and make people with disabilities feel reduced to their condition.

**Points for Discussion:**

- Why are questions about appearance or disability often inappropriate?
- How do peers’ reactions reinforce feelings of being “different”?
- What is the line between curiosity and disrespect?
- How can bystanders intervene supportively without being patronizing themselves?
- How should we teach children and students to approach difference with empathy?

**12. Speaking Over**

**Video Title:** *Stool at Work*
**Produced by:** South Australian Office of the Commissioner for Equal Opportunity
**Year:** 2021
**Language:** English
**Scene Description:** Roger, an employee with a back injury, is excluded from decisions about his own accommodation. Others discuss him without his input and discourage him from speaking up.
**Theme Alignment:** The clip shows how disabled individuals are often spoken about rather than spoken with, undermining their agency and autonomy.

**Points for Discussion:**

- Why is it problematic when others speak *about* a person with disability rather than *with* them?
- How does the secretary’s tone show assumptions about Roger’s abilities and rights?
- What power dynamics emerge when one party controls the conversation?
- How does the threat of “making things difficult” reflect structural discrimination?
- What does true inclusion look like in workplace accommodations?
