# Supplementary material for: Every Word and Action: Using Cinemeducation to Enhance Medical Students’ Awareness of and Upstander Responses to Disability Microaggressions
Source: MedEdPORTAL. 2026 Aug 14;22:11626. doi: 10.15766/mep_2374-8265.11626 (PMC13473049; doi:10.15766/mep_2374-8265.11626)
Supplement: Supplementary file 1 [file mep_2374-8265.11626-s001.zip › G. Self-Reflection Sheet.docx]

Student’s SELF REFLECTION

**What was happening in the scene?**
(Briefly describe the interaction or moment that stood out.)

*__________________________________________________________________________*
*__________________________________________________________________________*
*__________________________________________________________________________*

**Which form of microaggression or bias did this clip illustrate?**
*______________________________________________________________________________*

______________________________________________________________________________

**How did you feel while watching this clip?**
*______________________________________________________________________________________________________________________________________________________________*
*_______________________________________________________________________________*
*_______________________________________________________________________________*

**Did it remind you of something you have seen, experienced, or done in the past?**
(If yes, briefly explain.)

*_______________________________________________________________________________*
*______________________________________________________________________________________________________________________________________________________________*
*_______________________________________________________________________________*

**What was the impact of the microaggression shown?**
(on the individual with disability and/or others in the scene)

*______________________________________________________________________________*
*______________________________________________________________________________*
*______________________________________________________________________________*

**What could have been done differently by the characters involved?**

*_______________________________________________________________________________*
*______________________________________________________________________________________________________________________________________________________________*
*_______________________________________________________________________________*
