# Supplementary material for: Every Word and Action: Using Cinemeducation to Enhance Medical Students’ Awareness of and Upstander Responses to Disability Microaggressions
Source: MedEdPORTAL. 2026 Aug 14;22:11626. doi: 10.15766/mep_2374-8265.11626 (PMC13473049; doi:10.15766/mep_2374-8265.11626)
Supplement: Supplementary file 1 [file mep_2374-8265.11626-s001.zip › H. Feedback Questionnaire.docx]

**Cinemeducation for Addressing Microaggressions & Disability Inclusion**

Note: Should be used at the end of the session.

**Dear Learner,**

Thank you for your active participation in the cinemeducation session. Your contribution made the session lively and meaningful. We hope you had an effective learning experience.

Kindly rate the session on a scale 5 point Likert scale and provide your valuable feedback to help us improve.

1 = Strongly disagree
2 = Disagree
3 = Neutral
4 = Agree
5 = Strongly agree

1. **The instructions provided prior to the activity were clear**

1 = Strongly disagree
2 = Disagree
3 = Neutral
4 = Agree
5 = Strongly agree

2. **The use of film clips made the session more engaging and relatable**

1 = Strongly disagree
2 = Disagree
3 = Neutral
4 = Agree
5 = Strongly agree

3. **The chosen clips effectively portrayed key themes related to microaggressions & disability inclusion**

1 = Strongly disagree
2 = Disagree
3 = Neutral
4 = Agree
5 = Strongly agree

4. **The self-analysis reflection sheet provided a personal space to write my emotions**

1 = Strongly disagree
2 = Disagree
3 = Neutral
4 = Agree
5 = Strongly agree

5. **The session helped me to recognize my personal biases**

1 = Strongly disagree
2 = Disagree
3 = Neutral
4 = Agree
5 = Strongly agree

6. **The session content was applicable to my personal life as well as future clinical practice**

1 = Strongly disagree
2 = Disagree
3 = Neutral
4 = Agree
5 = Strongly agree

7. **The session helped me improve my understanding of disability inclusion**

1 = Strongly disagree
2 = Disagree
3 = Neutral
4 = Agree
5 = Strongly agree

8. **The session was organized well with proper time management**

1 = Strongly disagree
2 = Disagree
3 = Neutral
4 = Agree
5 = Strongly agree

9. **The facilitator created a safe, inclusive, and engaging space for discussion.**

1 = Strongly disagree
2 = Disagree
3 = Neutral
4 = Agree
5 = Strongly agree

10. **The group discussion allowed for diverse perspectives to be shared respectfully**

1 = Strongly disagree
2 = Disagree
3 = Neutral
4 = Agree
5 = Strongly agree

11. **I felt emotionally safe and supported during the session**

1 = Strongly disagree
2 = Disagree
3 = Neutral
4 = Agree
5 = Strongly agree

12. **I would recommend this session to other medical students**

1 = Strongly disagree
2 = Disagree
3 = Neutral
4 = Agree
5 = Strongly agree
